# Analysis of an international collaboration for capacity building of human resources for eye care: case study of the college-college VISION 2020 LINK

**DOI:** 10.1186/s12960-017-0196-1

**Published:** 2017-03-14

**Authors:** Nyawira Mwangi, Marcia Zondervan, Covadonga Bascaran

**Affiliations:** 1 International Centre for Eye Health, London School of Hygiene & Tropical Medicine, Keppel Street, London, WC1E 7HT United Kingdom; 2 Kenya Medical Training College/Ministry of Health, Nairobi, Kenya

**Keywords:** Capacity-building/development, COECSA, Health links, Human resource for eye health, Partnership, Royal College of Ophthalmologists, VISION 2020 LINKS

## Abstract

**Background:**

There is an extreme health workforce shortage in Eastern, Central, and Southern Africa. Shortage of eye care workers impedes effective implementation of prevention of blindness programs. The World Health Organization has identified education, partnership, leadership, financing, and policy as intertwined interventions that are critical to resolving this crisis on the long term.

**Case presentation:**

The VISION 2020 LINK between the College of Ophthalmology of Eastern, Central, and Southern Africa and the Royal College of Ophthalmologists in the United Kingdom aims to increase the quantity and quality of eye care training in East, Central, and Southern Africa through a focus on five strategic areas: fellowship examination for ophthalmologists, training the trainers, curriculum development for residents in ophthalmology and ophthalmic clinical officers, continuous professional development (CPD), and mentoring program for young ophthalmologists. This study examined how education and partnership can be linked to improve eye care, through an evaluation of this north-south link based on its own targets and established frameworks to guide north-south links.

**Methods:**

An exploratory qualitative case study design was used. Twenty-nine link representatives were recruited through purposive sampling and snowballing. Face-to-face interviews were conducted using a semi-structured interview schedule that incorporated the components of a successful link from an existing framework. Documents pertaining to the link were also examined. Thematic analysis was used for data analysis.

**Results:**

The findings revealed that the perception to the contribution of the link to eye care in the region is generally positive. Process indicators showed that the targets in three strategic objectives of the link have been achieved. Framework-based evaluation also showed that the link is successful. Mutual learning and development of friendships were the most commonly identified success factors. Inadequate awareness of the link by college members is a key challenge.

**Conclusion:**

The study concludes that the link is active and evolving and has achieved most of its targets. Further developments should be directed to influence health system strengthening in Eastern, Central, and Southern Africa more strategically. The study recommends expansion of the scope of collaboration to involve multiple health system building blocks.

## Background

The World Health Report [1] demonstrated the huge scale of the global health worker shortage. It is a crisis that many low- and middle-income countries face. Thirty-six of these countries are in Africa, including the countries that are members of the College of Ophthalmology of Eastern, Central, and Southern Africa (COECSA) [1]. Although each of the COECSA countries is unique, they collectively face a shortage of eye health workers in terms of absolute numbers, skills, and distribution.

The World Health Organization (WHO) human resource for health action plan [2] identifies education, partnership, leadership, financing, and policy as five interlinked interventions that will contribute to resolving the health workforce crisis. Long-term north-south health links between developed and developing countries represent international partnership. Links are often dedicated to a specific intervention, such as capacity building in Africa or research collaboration.

Health links are formalized long-term voluntary partnerships between counterpart health organizations in the United Kingdom and developing countries (DC), whose primary purpose is to build capacity in the DC through targeted training [3]. The number of health links is increasing rapidly; there were at least 130 links between the United Kingdom and developing countries [3] in 2013. Many of them focus on capacity building. This is in line with the Crisp Report [4] which highlighted the role of the United Kingdom in scaling up health workers’ education, training, and employment in developing countries.

The VISION 2020 LINKS program was launched in 2004 by the International Centre for Eye Health, for the purpose of capacity building for eye care in Africa [5]. This program runs within the VISION 2020 strategy for the elimination of avoidable blindness by the year 2020. There are 27 such LINKS in 15 African countries, and one in Indonesia. Most of them involve hospital-to-hospital twinning. The link between COECSA and the Royal College of Ophthalmologists (RCOphth) in the United Kingdom is a unique college-to-college VISION 2020 LINK.

A few studies have investigated the contribution made by health links [3, 6–11]. However, there is still paucity of evidence regarding this complex phenomenon. This continues to be the case despite the Kampala Declaration [12] which stressed the need to collect evidence on interventions for the health workforce. The Crisp Report [4] also emphasized the need to evaluate partnerships so as “to understand what works, where and why.”

As the need to examine the success of links has gained prominence, frameworks for such an evaluation have also been developed. There are three published frameworks, all specifically designed to evaluate United Kingdom-Africa partnerships. The *National Health Service* (NHS) [13] describes five key principles, which are derived from the Paris Declaration for Aid Effectiveness [14] and the subsequent Accra Agenda for Action [15]. The *Africa Unit* [16] has in turn identified a total of 10 principles. These two frameworks describe general principles that guide health links. In contrast, the framework by Eastwood et al. [17] listed 13 specific components that drive the success of a health link; thus, it was used for the study. Tables 1, 2, and 3 outline the criteria used in each framework. The aim of the study was to evaluate the progress made in the strategic objectives of the COECSA-RCOphth LINK during its implementation and to measure its success using an established health link evaluation framework.

**Table 1:** Components of a successful link (Eastwood et al.)

1. Regular personal contact between individuals from both groups
2. Mutually agreed, locally relevant aims: clinical priorities, teaching programs, research objectives, staff development, or a mixture of all four
3. Clear written guidelines, preferably at the institutional level as to how the link will be fostered and supported
4. Acceptance that the scope of the collaboration will not be limited by the priorities of funding agencies in developed countries
5. Bilateral annual visits
6. Vision of a long collaboration (>10 years), not a transient one
7. Training of individuals from developing countries taking place in their own country as much as possible
8. Ability to respond to a changing environment at either end
9. Involvement of nurses and other health workers in the collaboration
10. Consider honorary contracts for individuals taking in established links; these can strength the links
11. Early planning as to means of securing future funding
12. Independence from commercial sources of funding, avoiding possible future conflicts of interest and maintaining ability to publish the results of research
13. Foster the development of other links and contribute to an international philosophy of links in general

**Table 2:** NHS principles of effective development assistance

1. Alignment to government or hospital health plans	
2. Harmonization with initiatives from other partners	
3. Evidence base with proper monitoring and evaluation	
4. Sustainability through long-term commitment by partners	
5. Mutual accountability with shared responsibility

**Table 3:** Principles of effective partnership (Africa Unit)

1. Shared ownership of the partnership	
2. Trust and transparency among partners	
3. Understanding each partner’s cultural environment and working context	
4. Clear and agreed division of roles and responsibilities	
5. Effective and regular communication between partners	
6. Strategic planning and implementation of partnership plan and projects	
7. Strong commitment from junior and senior staff and management	
8. Supportive and enabling institutional infrastructure	
9. Systematic monitoring and evaluation of partnership and partnership projects	
10. Sustainability	

## Case presentation

The LINK was launched in 2008 by a memorandum between the East African College of Ophthalmologists (EACO), which is the predecessor to COECSA, and the Royal College of Ophthalmologists, United Kingdom. COECSA was subsequently formed in 2012 upon the merger between EACO and the Ophthalmological Society of Eastern Africa (OSEA). The COECSA-RCOphth VISION 2020 LINK aims to increase the quantity and quality of eye care training in Eastern, Central, and Southern Africa through a focus on five strategic areas: fellowship examination for ophthalmologists, training the trainers, development of harmonized curricula for training residents in ophthalmology and ophthalmic clinical officers, online continuous professional development (CPD) program, and a mentoring program for young ophthalmologists. The LINK is supported by a grant from the Department for International Development (DFID) through the Tropical Health Education Trust (THET).

The eight countries involved in the LINK at the time of the study were Burundi, Ethiopia, Kenya, Malawi, Rwanda, Tanzania, Uganda, and Zambia. All the countries face a severe shortage of health workers, including eye health workers. Training programs for ophthalmologists and ophthalmic clinical officers are already established in all the countries except Rwanda and Burundi. The training programs, however, are heterogeneous in curriculum content and delivery. The first phase of implementation of the LINK was from 2010 to 2012, and it was initiated with three ECSA countries (Kenya, Uganda, Tanzania). The second phase was 2013–2014 and involved eight countries. Additional countries plan to join COECSA in the near future, such as South Sudan.

## Methods

An extensive literature search was conducted to provide an in-depth understanding of health links. In addition, preliminary discussions were held with the COECSA president and with the VISION 2020 LINKS manager to gain an insight into the genesis of the COECSA-RCOphth LINK. Subsequently, a study protocol was developed for a qualitative case study. The study was designed in accordance with the principles of the Helsinki Declaration [18] and the Belmont Report [19]. The London School of Hygiene & Tropical Medicine granted ethical approval while the Sear Family Foundation provided funding for the study.

Data was collected between May and July 2014. Twelve LINK coordinators (one from each ECSA country and three from the United Kingdom) were initially identified from the VISION 2020 LINK office (purposive sampling) and contacted by email to arrange for face-to-face interviews. Nineteen additional link representatives were identified at these interviews through snowballing (Tanzania 2, Uganda 4, Malawi 2, Zambia 1, Kenya 4, Ethiopia 1, United Kingdom 3, and COECSA secretariat 2). Semi-structured interviews were conducted in the United Kingdom and in ECSA. The main topics covered in the interview were the implementation of the LINK, contribution of the LINK to capacity building in ECSA, and the progress in the strategic objectives. The framework for evaluation of links proposed by Eastwood et al. [17] was incorporated in the interview schedule. The ethos of this framework is that success of a link depends on how effectively it is run. This framework identifies 13 components of a successful north-south link between the United Kingdom and developing countries (Table 1). It provides criteria for determining the likelihood that a link will be successful in the long term.

Interviews were conducted in English and audio recorded. Participants gave written informed consent. Link-related documents were also examined to provide triangulation. Data analysis was done using thematic analysis.

## Results

Twenty-nine of the 31 people contacted consented and were available for interview (94% response rate). The other two consented but were unavailable due to competing commitments. Both of them were from COECSA, one was an ophthalmologist while the other was a manager. Twenty-three of those interviewed (79% of respondents) were ophthalmologists while the other 21% were managers and non-ophthalmologist health workers. Seventeen per cent were from the United Kingdom while 83% were from ECSA region. The majority of the participants had been involved in both phase I and phase 2 of this LINK. However, their period of involvement ranged from 1 year to the entire period of the LINK.

### Contribution of the link to human resource capacity development

**Fig. 1:**
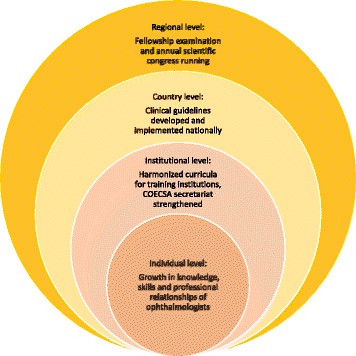
Themes contributing to capacity-building

Participants generally perceived the LINK positively and identified themselves, peers, institutions, or countries as beneficiaries of the LINK. The benefits were in the form of training, examinations, workshops, or exchange visits to the partner country. Link documents (reports) that were examined captured similarly positive feedback from residents and ophthalmologists regarding improved teaching and examination methods. Several themes related to capacity development were identified and were classified into three levels: personal, institutional, and country and regional level (Fig. 1). At the individual level, knowledge and skills of ophthalmologists had been improved through various kinds of training, including leadership, curriculum development, guideline development, and training as examiners for the fellowship examination. The strength of relationships with mentors and peers had also increased leading to enhanced professional trust.

At the institutional level, training institutions for Master of Medicine (M.Med; residency) and ophthalmic clinical officers (OCO) have had harmonized curricula developed. The trainers for M.Med have also received training in teaching and assessment skills under the “train the trainers” (TTT) program. New assessment methods have been adopted such as the objective structured clinical examination (OSCE) to replace the traditional long and short case method. The structured viva examination method has been maintained.

At the country level, capacity has been developed in the areas of clinical guideline development, curriculum development, and examiner training. At the regional level, continuous professional development (CPD) occurs at the annual scientific congress. The fellowship examination runs annually, and a total of 31 ophthalmologists had taken it by 2013. Thirty-seven examiners for this examination have also been trained, 36 from the eight countries (Rwanda 2, Kenya 10, Malawi 3, Zambia 3, Tanzania 3, Uganda 11, Ethiopia 3, Burundi 1) and one from South Sudan which has intention to join COECSA.

The perception of the participants to the contribution of the LINK was generally positive. For example:

My overall opinion is that the LINK is going well and should continue. Although for us from (names country) the membership in COECSA is very new, we have learnt that working together brings change (COECSA participant)

It is rewarding to see that much progress has been made and there are tangible outputs (United Kingdom participant)

### Progress in the strategic objectives of the LINK

#### Objective 1: to train examiners and provide supervision for the fellowship examination

The fellowship examination began in 2010, and the number of candidates registering increased progressively (Table 4). Thirty-seven examiners for the fellowship examination were trained by 2013, with 26 having been trained in phase 1 (from five institutions in Kenya, Uganda, and Tanzania) and 11 (from all eight countries) being trained in 2013 of the LINK. There were plans to train 26 more examiners in 2014.

The fellowship examination started with the LINK in 2010. It has run every year since then, and we now have a pool of COECSA examiners (COECSA participant)

**Table 4:** Progress in fellowship examination

Phase 1 (Jan 2010–Dec 2012) achievements	Phase 2 (Jan 2013–2014) achievements
- Examination started in 2010 and ran annually - 20 fellows examined - 26 examiners from 5 institutions trained - 2 RCOphth exam facilitators participated in the examination annually - Draft examination manual prepared - Examination framework developed - 6 COECSA delegates observed Part 11 RCOphth examination	- Examination ran annually - 11 ophthalmologists from 8 countries took the examination in 2013, 30 ophthalmologists scheduled to take the exam in 2014 - 11 examiners trained in 2013 - 2 RCOphth representatives participated in each COECSA exam - Examiners manual prepared - 4 COECSA ophthalmologists observed the Part 11 exam in United Kingdom in 2013 and 2014

#### Objective 2: to train the trainers (TTT) in teaching and assessment methods

Trainers in the training institutions have been trained through the LINK (Table 5). The training of the trainers targeted to train 16 trainers by 2013 and an additional eight in 2014. The distribution of those trained by 2013 by country was Uganda 4, Tanzania 2, Ethiopia 1, Rwanda 2, Burundi 1, Zambia 1, Malawi 1, and Kenya 4 (total 16). The imbalance in country representation was noted by the partners and would be corrected in the subsequent training. Eight more were to be trained in 2014.

**Table 5:** Progress in training the trainers

Phase 1 (Jan 2010–Dec 2012) achievements	Phase 2 (Jan 2013–2014) achievements
- Clinical guidelines for glaucoma developed - Faculty trained on student assessment methods - Residency examination methods changed from long case and short cases to OSCE - Residency theory exams adopted the model of multiple choice and constructed response answers - 30 participants trained in research methodology	- 2 train the trainer (TTT) courses held; 16 participants trained on methods for training - 19 participants trained on development of clinical guidelines - Working groups for guideline development for *biometry*, *retinoblastoma*, *allergic conjunctivitis*, *diabetic retinopathy*, and *glaucoma* established and running

The participants felt that this training had changed practice in teaching and assessment:

Exams are the shining signature of the LINK (COECSA participant)

Or approach to assessment of students in residency has changed drastically, and the assessments are now more objective…previously examinations were conducted inappropriately but we changed following many trainings that were facilitated by RCOphth (COECSA participant)

The examination area was seen to be the most successful among the five objectives. One of the reasons attributed to this success was the enthusiasm of the examination leads, which gave this objective “a big driving force” (RCOphth participant).

#### Objective 3: to harmonize training curricula for ophthalmologists and ophthalmic clinical officers

The LINK’s target was to develop two new curricula (ophthalmology residency and OCO curricula; (Table 6). In the first phase of the LINK (2010–2012), 16 trainers from Kenya, Uganda, and Tanzania were trained in curriculum development. In the second phase of the LINK, two curriculum development workshops for both residency and OCO curricula were held as planned. Fifteen ECSA representatives from training institutions participated in the residency curriculum (Rwanda 1, Uganda 2, Kenya 4, Tanzania 2, Malawi 2, Burundi 1, Zambia 1, and Ethiopia 2). Eight representatives participated in the OCO curriculum development. The two countries without training programs, Burundi and Rwanda, will be able to use the curricula to develop their own training programs in the future. The OCO curriculum was completed and validated while work on the residency curriculum was in progress. Participants expressed that there had been good progress in this objective:

Curriculum development has helped us to set standards in COECSA through use of a harmonized curriculum (COECSA participant)

**Table 6:** Progress in curriculum development

Phase 1 (Jan 2010–Dec 2012) achievements	Phase 2 (Jan 2013–2014) achievements
- 16 trainers trained in curriculum development - M.Med residency curriculum standardized - Curriculum development for OCO program commenced	- 8 trainers participated in OCO curriculum workshops - 15 trainers participated in curriculum workshops for the residency program - Draft residency curriculum document prepared OCO curriculum validated by stakeholders - Flexibility maintained in the curricula in view of heterogeneity of training programs in the different training institutions

#### Objective 4: to develop online continuous professional development for ophthalmologists and OCOs

Although some progress had been made towards making CPD available in the region, online CPD was not available (Table 7).

**Table 7:** Progress in development of CPD program

Phase 1 achievements	Phase 2 achievements
- 13 COECSA participants attended a workshop on CPD development - CPD framework prepared - CPD meetings held monthly - OSEA congress held annually	- 1st COECSA congress held in 2013 - COECSA accredited as a CPD provider in Kenya - Content from 9 CPDs published on COECSA CPD system - Online CPD platform not yet implemented

We have some CPD activities but they are not accessible to everyone (COECSA participant)

I am not sure how far we are with the plans for online CPD, but we need to have a platform for it, and to develop our own online resources in future (COECSA participant)

There was significant inequity in the availability of CPD opportunities, with the ophthalmologists who work in the cities and in training institutions being advantaged and those in rural areas remaining disadvantaged. The most common reasons given for delay in implementing online CPD was the high cost of equipment and software, the lack of a CPD strategy, and the difficulty in entrenching the CPD concept.

#### Objective 5: to develop a mentoring program for newly qualified ophthalmologists

Progress in this objective mainly occurred in phase 2 of the LINK, during which the Young Ophthalmologists Forum (YOF) held two forums and 24 ophthalmologists received leadership training. The leadership training, and the motivation arising from it, was the key reference made in relation to this objective:

I am not young but one of my colleagues attended the first Young Ophthalmologists Forum at Kigali in 2013 and another meeting in Dar…and is now extremely motivated, …even taking up leadership positions here(COECSA participant)

Although some participants indicated that young ophthalmologists felt alienated and lacked opportunities for participation, other participants did not concur:

It depends on how actively the individual demonstrates readiness to be involved. I am a young ophthalmologist and I am fully involved. I have not experienced any lack of opportunity and I have not heard of any complaint (COECSA participant)

### Success of the LINK

Table 8 shows the assessment of the success of the LINK against the Eastwood et al. [17] criteria. The LINK fully satisfies 10 of the 13 criteria.

**Table 8:** Success of the LINK based on the components of a successful link [23] (Eastwood et al., 2005)

1. *Regular personal contact between individuals from both groups* Occurs through curriculum workshops, TTT courses, annual fellowship examinations, and annual scientific congress*.*
2. *Mutually agreed, locally relevant aims: clinical priorities, teaching programs, research objectives, and staff* Are articulated as the 5 strategic objectives in the memorandum of understanding and there are targets for each phase of the grant.
3. *Clear written guidelines, preferably at the institutional level as to how the link will be fostered and supported* Are in the form of the VISION 2020 LINKS TOOLKIT. This is a general tool for all overseas partners. Although it is available online, most participants were not aware of it. There is also a memorandum of understanding signed at the launch of the LINK, whose content most participants were not conversant with.
4. *Acceptance that the scope of the collaboration will not be limited by the priorities of funding agencies* The scope of collaboration is determined by the needs of COECSA and this is indicated in the MOU.
5. *Bilateral annual visits* Occur in the context of observing exams and attending annual congress in both United Kingdom and COECSA.
6. *Vision of a long collaboration (>10 years), not a transient one* The LINK is envisaged to be long term, although the actual duration is not explicitly stated. It is to continue “as long as the partners wish to collaborate” according to the MOU.
7. *Training of individuals from developing countries taking place in their own country as much as possible* All training courses and workshops that are meant to achieve the strategic objectives occur in COECSA region
8. *Ability to respond to a changing environment at either end* Is demonstrated by the setting up of committees as organs of decision-making and use of effective communication. The decision to postpone COECSA congress 2014 in view of the Ebola outbreak in West Africa during that period exemplifies this.
9. *Involvement of nurses and other health workers in the collaboration* This has not been adequately achieved and participants reported the need for it: “You will note that COECSA is college of ophthalmology…not really college of ophthalmologists…so we should involve more cadres. We want to begin with the colleges that train the mid-level cadres” (COECSA participant)
10. *Consider honorary contracts for individuals taking in established links; these can strength the links* This has not been achieved; facilitators usually work on a volunteer basis.
11. *Early planning as to means of securing future funding* This has been pursued and achieved. A participant expressed: “We are alert to find out when the next round of funding is around the corner, and we put in (submit to funders) our proposal early”
12. *Independence from commercial sources of funding, avoiding possible future conflicts of interest* There has not been any funding from commercial sources. However, one participant suggested that: “Funding from suitable commercial sources such as equipment manufacturers who have an education component may not present a high risk for conflict of interest and may be considered in the future”
13. *Foster the development of other links and contribute to an international philosophy of links in general* The LINK is established in the context of 27 other VISION 2020 LINKS and supports the establishment of additional twinning LINKS in the foreseeable future. It is complementary to other links such as the Munich link without duplication or conflict.

### Perceived benefits of the LINK

There was marked congruence in the perception on the benefits of the LINK, as identified by partners from both the United Kingdom and ECSA.

Participants referred to increase in personal and institutional capacity, development of friendships and networks, cross-cultural awareness, motivation to change practice of training, increased teamwork, raising the profile of ophthalmology, and the opportunity for mentoring relationships. However, the most commonly identified benefits were shared learning and development of friendship:

It is about sharing experiences and learning … it is not just copying directly from RCOphth and replicating it in COECSA (United Kingdom participant)

Support and friendship is very good…now when I get an email from (name of a counterpart in COECSA) I can identify with the situation as they express it … this is because I have the experience of that context now (United Kingdom participant)

For me the LINK has created professional space …I am inspired to work even though resources are limited (COECSA participant)

The profile of the college (COECSA) and training institutions has been raised. In Kigali congress we had ministerial presence and this means there is recognition (United Kingdom participant)

COECSA members also cited the benefit of affiliate membership to RCOphth being access to journals and CPD materials from RCOphth.

### Challenges inherent to the LINK

Like the benefits, the challenges did not show variation by collaborating partner or by country. However, one challenge that was articulated in only one ECSA country is the lack of involvement of ophthalmologists in private practice in LINK activities. Participants from the seven other ECSA countries indicated that in their countries, those in private practice were fully involved or that in some countries, no ophthalmologists were exclusively engaged in private practice.

Inadequate awareness of the LINK by college members in both the United Kingdom and ECSA was the leading recognized challenge. In addition, the multi-country nature of the partnership also carries some unease:

Keep in mind that the broader the link, the more the parties in the partnership, a kind of scramble results and you may not get what you wish. This does not occur when you are dealing with a specific hospital to hospital link, as you are not competing with others in the team (COECSA participant)

The other challenges are listed in Table 9. Contextual factors and resource constraints were identified by both partners. However, insufficient involvement in various aspects of the LINK and the challenge of multiple actors were mainly identified by the ECSA partners.

**Table 9:** Challenges encountered in the link

Challenges identified by COECSA partners	Challenges identified by United Kingdom partners
**Challenges in the context of the collaboration framework** -Lack of opportunity for clinical participation in the United Kingdom during visits (only observership) -Delays in obtaining visas for travel to the United Kingdom for LINK visits -Institutional bureaucracy delays changes such as adoption of curricula **Challenges due to multiple actors in the collaboration** -Harmonization is complex due to the multi-country nature of COECSA -Heterogeneity of training programs impedes a uniform curriculum -“Scramble for opportunities” means countries and institutions have not benefited equally **Resource constraints** -Inadequate time for LINK work -Funding is unpredictable -Delays in release of funds for scheduled activities **Inadequacy of involvement** -Ophthalmologists outside cities and teaching hospitals are not actively involved -CPD inaccessible to those working outside the large cities -Inadequate involvement of ECSA countries in agenda setting leading to insufficient ownership	**Challenges in the context of the collaboration framework** - COECSA has not taken full ownership of LINK activities which creates gaps in running activities -Cultural differences -Leadership gaps in COECSA -Reliance on a few individuals to carry out key roles both in the United Kingdom and COECSA -CPD concept has been difficult to entrench -Inadequate communication of decisions or changes related to LINK activities **Resource constraints** -Time constraints as United Kingdom partners have to use their personal time for LINK activities -Uncertainty of funding -Lack of appropriate technology in COECSA

### Suggestions for improvement

Participants identified the areas of improvement in the LINK. While the specific suggestions by the north and south partners differ, they cut across the phases of planning, implementation, and evaluation of the LINK (Table 10).

**Table 10:** Suggestions for strengthening the link

Suggestions from COECSA partners	Suggestions from United Kingdom partners
*Program planning and design* • There is need for continuous dialog and joint planning • COECSA to drive the process by demonstrating both demand and ownership • More opportunities for visits to the United Kingdom to “see how things are done” • LINK to facilitate setting up of more VISION 2020 twinning links in the region • LINK to provide more scope for interaction between the institutions in the ECSA region • Expand the scope of collaboration to include research, subspecialty programs, mentoring and instruments *Program implementation* • Build capacity of editors of the scientific journal • Involve middle-level eye care cadres in the LINK • Training of examiners should be more intensive than just 1 day before the exams, “perhaps even organized in modules” • Make the partnership equal and mutual • Strengthen leadership of the COECSA and the LINK *Evaluation* • Gather evidence to demonstrate impact of the LINK • Strengthen monitoring and evaluation, and source funding for it	*Program planning and design* • Build on the successes of the partnership in future planning *Program implementation* • COECSA needs to build develop staff to take lead in all the activities • Ensure the right people are on board for LINK activities • Encourage continuity for lead roles or smooth handover/transition when new persons take up roles • Aim to make each activity effective and evaluate effectiveness • Ensure United Kingdom facilitators have clarity on their roles • Improve frequency and regularity of communication *Evaluation* • COECSA to strengthen reporting on LINK activities • Constantly evaluate if expectations of COECSA are being met

## Discussion

Resolution WHA 59.23 of the World Health Assembly (2006) focuses on scaling up of health worker production. It calls on both developed and developing countries to engage urgent, sustained, and innovative action to address this crisis. Such action includes capacity building for the health workforce. This need is a motivating factor for institutions to engage in strategic institutional, national, regional, and international collaboration. The Baguley et al. study [7] and the Crisp Report [4] pointed to the increase in capacity in training and research as one of the favorable outcomes of health links particularly in developing countries. This was a broad study that evaluated the achievement of the targets in the five strategic objectives of the LINK, the general contribution of the link to capacity development, and the success of the LINK. It provides evidence for the extent to which the LINK has addressed training needs in ECSA. It also gives insight into the applicability of existing frameworks for guiding health links to the context of multi-county partnerships.

The main shortcoming of the qualitative study methodology is the potential for response, reporting, and social desirability bias. We tried to address this by involving participants from both the United Kingdom and ECSA partners to provide a balanced view. Triangulation of sources of data also ensured internal validity. The evaluation of the success of the LINK was limited to a focus on the way the LINK is run, as health impact or economic evaluation were beyond the scope of the study. Although the case study is a unique health link, comparability of findings with other links is feasible because specified frameworks for evaluation were used.

### Capacity development

Capacity has been built at micro, meso and macro levels. The first phase of the link concentrated on building individual capacity of trainers and of training institutions for M.Med residency and OCO training. The second phase was marked by a transition from institutional to country focus, and the number of countries in the LINK has increased from three to eight. Skills development has been the main avenue for building capacity. The specific skills have included leadership, teaching, assessment, and curriculum and clinical guidelines development, a feature that is also noted in other links [11].

The LINK achieved the targets that had been set for three of its five objectives, namely fellowship examination, training the trainers, and curriculum development. One of the factors considered responsible for the success is in the area of examinations is that the personalities at the lead in ECSA have been a strong driving force, by virtue of demonstrating strong leadership and commitment to agreed goals. This is not a unique finding as other studies have shown that such individuals contribute significantly to initiating links or making them successful [8, 20]. The corresponding challenge, which was identified in the LINK, is to ensure continuity of achievement even after these individuals have handed over to others or moved on in their careers [7]. It also suggests that leadership training is vital in health links. Documenting the progress made in links through a study like this one may also contribute to preserving institutional memory and motivating future leaders of the LINK. The importance of commitment to agreed goals has also been identified in the health links literature. DFID, in its evaluation of north-south partnerships in countries such as Malawi, Tanzania, and Uganda, highlighted the importance of measuring progress towards an agreed work plan. This might involve proxy indicators for capacity building such as number of staff trained [8].

Similarly, although only two forums for leadership training for young ophthalmologists had been held, it had generated a lot of motivation and also willingness to take up new roles. Viewed from the concept of health system strengthening, the LINK has influenced leadership capability, which relates to the stewardship or governance building block of health systems. [21] Some participants also expressed that the leadership training contributed to succession planning as new leaders were prepared to take lead roles. It reinforces what other studies have found, that succession planning should not be neglected during capacity building [20].

The LINK was on target in the curriculum development objective. It is worth noting that the two curricula were developed jointly by the partners, and not just replicated from RCOphth. This ensures that the curriculum is relevant to the COECSA context. Previous research on health links between the United Kingdom and Africa provides evidence that failure to take this precaution may render a curriculum irrelevant to the intended user [7].

Although the residency and OCO training programs in COECSA are heterogenous in terms of organization and duration, the new harmonized curriculum can be applied across the institutions. It allows for flexibility and meets the needs of diverse COECSA institutions. Training institutions can adopt the curriculum in totality or adopt portions of it, subject to a set minimum criteria, which gives flexibility to member institutions. Only one paper has previously explicitly documented the importance of flexibility and creativity in partnerships [22].

Continuing professional development is an important intervention for building capacity in human resource for health. Peck et al. [23] have defined it as

The process by which health professionals keep updated to meet the needs of patients, the health service, and their own professional development. It includes the continuous acquisition of new knowledge, skills, and attitudes to enable competent practice

Online CPD had not yet been implemented in the LINK as targeted and access to regular CPD remained very variable. Ophthalmologists working in rural areas and outside teaching institutions were relatively disadvantaged, yet they see the bulk of the patients and are also more prone to professional isolation. This is a mismatch between the availability of CPD and the pattern of the need for it. The LINK must strive to break the mismatch rather than aggravate it, for links have been known to build disparities [7]. The hindrances to the CPD agenda, such as difficulty in entrenching the CPD concept, have also been documented in other studies [6].

Progress regarding the Young Ophthalmologists Forum occurred in phase 2 of the partnership, and 24 young ophthalmologists benefited from leadership training. Although the mentoring program was still being developed, participants reported that the leadership training had generated motivation (professional inspiration). Motivating young ophthalmologists might help to avoid decline in enthusiasm as the partnership proceeds, a potential risk that was identified in the initial grant for the LINK. Mentoring young ophthalmologists, together with access to subspecialty training as participants suggested, has potential to impact both the service delivery and the leadership and governance building blocks of health systems. This is an important avenue for the LINK to go beyond focusing on just one building block, the health workforce (capacity building).

### Benefits and challenges

The benefits, challenges, and suggestions identified in this study are wide, and they align to the findings in other literature [7, 8, 10, 20]. The commonest benefit cited by ECSA participants was capacity development while United Kingdom participants cited motivation from the experience of different cultures and eye care in low-resource contexts. Two beneficial aspects that were invariably expressed by participants from both partners are the focus on *mutual learning* and the *development of friendship* with counterparts, leading to personal satisfaction [10]. Although the study was not designed to investigate the precise configuration of these friendships, the findings suggest that links have roles beyond transfer of skills. Strong personal relationships have similarly been found to be a positive factor in links between Wales and Africa [10]. They may promote professional trust, interaction, and shared learning. The implication is that links should promote the securing of these benefits.

The LINK faces a number of challenges (Table 8), most of which can be addressed or minimized by the LINK partners. Most of the challenges are not unique to this LINK, but their presence shows that links can generate some frustration [22]. Although participants indicated that the LINK had helped to raise the profile of ophthalmology and of training institutions, the awareness of the LINK among members of the two colleges is still insufficient. Members who do not have direct responsibilities in running the LINK lack awareness on the role of the LINK. As one participant said, there is “need to do the fanfare.” The partners thus need to raise the profile of the LINK in college community. Furthermore, imbalance in the cumulative number of beneficiaries of the LINK from each of the eight countries was evident. The imbalance was in favor of the countries that have been part of the LINK for a longer duration or have had longer experience with ophthalmology training. They have a higher cumulative number of training institutions and ophthalmologists, who are more likely to participate in the LINK activities. However, the VISION 2020 LINK office had plans to build balance in subsequent activities.

Some literature documents that building relationships, especially in the early stages of partnership, is the most daunting and time-consuming challenge to partnerships [22]. This would have been anticipated in this multi-country collaboration. In contrast, this study found no problems with building relationships. One possible explanation is that the LINK has existed for several years, and it may have already overcome relationship difficulties that occur during the formation of links. However, competition for opportunities among the countries was reported, which relates to the multi-country nature of the partnership and the resource constraints that are an added challenge. Contextual factors such as cultural differences and policy concerns (delays in travel visas and lack of opportunity for ECSA partners to participate in clinical care in the United Kingdom) were also articulated in this LINK. This is a reflection of the diversity in the environment that the partners operate in.

### Success factors

Success of a link can be assessed from many angles. This LINK can be considered successful based on the Eastwood et al. framework [17] and also on the frameworks by NHS and the Africa Unit (Table 9). Both northern and southern partners expressed great value and potential for increased collaboration, suggesting that the reputation of the LINK and the way it is run is good.

At the strategy level, the LINK was established through a memorandum of understanding that specified mutually agreed objectives. The LINK has also embraced joint planning, even in proposal writing for funding. Strategic planning is a best practice that is similarly articulated by the African Unit framework [16]. The vision is of a long-term collaboration. Training a pool of trainers and examiners who will cascade the training is a measure for *sustainability*, as the trainees will use this skill for the rest of the career in training and will train successors. However, it does not address sustainability of funding for LINK activities, which depend on grants. Sustainability is a principle that is also emphasized by the NHS [13] and African Unit [16] frameworks.

At the operational level, the link has embraced regular contact between individuals involved in the partnership, bilateral annual visits, and in-country training activities. These practices strengthen liaison, communication, local ownership, and commitment to the partnership. There is strong commitment to the LINK by the presidents and chief executives of the two colleges, deducible from their participation in activities such as the annual congress. Participants mentioned using personal time for LINK activities, which further implies commitment. There are LINK coordinators in each of the eight countries involved in the partnership and in each of the committees responsible for various activities such as curriculum development or examinations. These committees have shared leadership from both partners. This is a reflection of *mutual accountability and shared responsibility* which are also emphasized by the NHS [13]. DFID found that these best practices are critical in north-south partnerships between the United Kingdom and ECSA countries such as Malawi, Tanzania, and Uganda [8]. The African Unit [16] similarly emphasizes *ownership*, *shared roles*, *and strong commitment from junior and senior staff*.

Although the LINK has demonstrated a level of achievement of ownership, participants from both partners also identified insufficient ownership as a challenge. This might suggest that there are different domains of ownership, and only a part of them have been satisfied, but this study did not seek to investigate these domains.

A strong *institutional infrastructure* is not visible in COECSA as it is a “young college” that is still developing its structures. The need for strengthening *monitoring and evaluation* was raised in the suggestions for improvement (Table 10), including the need to source for funding for it. This suggests that one of the factors that may determine the success of links is availability of funding. The implementation of other best practices such as bilateral annual visits and honorary contracts for facilitators also depends on the funding. As unpredictability of funding was identified as a challenge in this LINK, it remains a potential threat to gains such as sustainability of the LINK or other best practices.

### Suggestions for improvement

The suggestions for strengthening the LINK (Table 10) reflect the potential for increasing collaboration and show that people recognize the value of the LINK and that it continues to be demand driven [4, 13]. The findings also suggest that the LINK is mutual between the northern and the southern partner, although not necessarily symmetrical, which is the inspiration for links [4, 9, 20] as they can leverage on a partner’s strength. There is recognition that south-south collaboration between COECSA countries themselves is currently minimal relative to the north-south collaboration and it needs strengthening. Eastwood et al. [23] document that successful links should foster development of other links; hence, the experience with this LINK might generate increasing interest in south-south links. Through intra-Africa collaboration, for example, centers of excellence in different sub-specialties can be established in different countries to serve the region.

## Conclusion and recommendations

This study provided evidence that this VISION 2020 LINK is active and evolving. Three targets were met while two remain to be completed. Continuity with the those objectives is desirable as they are demand driven and this study showed that they are achievable. The successes provide a beacon of enthusiasm, while additional emphasis can be given on those objectives that have not been achieved in full (CPD and mentoring for young ophthalmologists).

Expansion of scope of collaboration is the next step; COECSA participants expressed the need for expansion of collaboration to include joint research, subspecialty training within the region, training for the editorial team of the scientific journal, mobilization of resources and appropriate technology, and sourcing equipment for eye care. The link can foster additional north-south VISION 2020 hospital twinning links, which several hospitals are seeking. It can also foster south-south interdependency between ECSA countries as they can synergistically leverage on each other’s capabilities and goodwill.

Future research may include an evaluation of the impact of the LINK on the training of ophthalmologists, for example, through a survey of a cohort of newly graduated ophthalmologists. An impact of the LINK on prevention of blindness may also be appropriate, though attribution of effect to the LINK may be challenging. An economic evaluation would identify if the LINK is a cost-effective way of addressing the human resource challenges in the ECSA region.

## Abbreviations

COECSA: College of Ophthalmology of Eastern, Central, and Southern Africa
CPD: Continuous professional development
DFID: Department for International Development
EACO: East African College of Ophthalmology
M.Med: Master of Medicine
OCO: Ophthalmic clinical officer
OSCE: Objective structured clinical examination
OSEA: Ophthalmological Society of Eastern Africa
RCOphth: Royal College of Ophthalmologists
TTT: Train the trainers
WHO: World Health Organization
YOF: Young Ophthalmologists Forum
